# Provider experiences of the implementation of a new tuberculosis treatment programme: A qualitative study using the normalisation process model

**DOI:** 10.1186/1472-6963-11-275

**Published:** 2011-10-17

**Authors:** Salla Atkins, Simon Lewin, Karin C Ringsberg, Anna Thorson

**Affiliations:** 1 Health Systems Research Unit, Medical Research Council of South Africa, Cape Town, South Africa; 2 Division of Global Health, Karolinska Institutet, Stockholm, Sweden; 3 Global Health Unit, Norwegian Knowledge Centre for the Health Services, Oslo, Norway; 4 Health Promotion Research Group, Nordic School of Public Health, Gothenburg, Sweden

## Abstract

**Background:**

Tuberculosis (TB) is a major contributor to the global burden of disease. In many settings, including South Africa, treatment outcomes remain poor. In contrast, many antiretroviral treatment (ART) programmes are achieving high levels of adherence and good outcomes. The ART programme model for maintaining treatment adherence may therefore hold promise for TB treatment. Changing treatment models, however, requires an assessment of how staff receive the new model, as they are responsible for programme implementation. Using the normalization process model as an analytic framework, this paper aims to explore staff perceptions of a new TB treatment programme modelled on the ART treatment programme.

**Methods:**

A qualitative approach was used. Interviews and focus group discussions were conducted with clinic staff from five intervention clinics. Data were analysed initially using qualitative content analysis. The resulting categories were then organised under the constructs of the normalization process model.

**Results:**

Staff recounted a number of challenges with implementing the programme. Interviews and focus group discussions identified factors relating to the main categories of the normalization process model. The key issues hindering the normalisation of the programme within clinics related to the interactional workability, relational integration and skill-set workability constructs of the model. These included hierarchical relationships, teamwork, training needs and insufficient internalisation by staff of the empowerment approach included in the programme. Logistical and management issues also impacted negatively on the normalization of the programme at the clinics.

**Conclusion:**

The normalization process model assisted in categorising the challenges experienced during implementation of the intervention. The results suggest that issues remain that need to be resolved before the programme is implemented more widely. Considerable work is needed in order to embed the intervention in routine clinic practice.

## Background

Globally, over two million people die from tuberculosis (TB) each year [1]. In South Africa, the incidence rate of TB is one of the highest in the world (approximately 960 per 100000 in 2007), but it is estimated that only 70% of patients are successfully treated [1], despite the widespread use of directly observed therapy (DOT) to maintain treatment adherence. It is also estimated that approximately 73% of TB patients in South Africa are infected with HIV [1]. Providing integrated treatment for TB and HIV could result in the more efficient use of human resources and more convenient and effective care for people living with these illnesses. The differing models of treatment used for these two diseases do not, though, facilitate easy solutions to integrating treatment at community level, suggesting that a change in treatment models is needed to improve their compatibility.

The ART community treatment model, which is based on intensive treatment counselling and preparation; self-supervised therapy; community-based lay treatment support; and regular follow-up by health professionals [2] may have potential for the delivery of TB treatment. In South Africa, ART programmes achieve higher treatment adherence in selected settings (over 80% at six months [3,4]) than DOT programmes (approximately 60% in South Africa overall [5]). Some of the reasons for poor adherence to TB treatment could include access to health care services; treatment side effects; beliefs about treatment and motivations to take it; conflicting priorities, for example the need to earn a living; discrimination and stigma; and social pressures not to take medication [6]. In addition, systematic reviews have contested the effectiveness of DOT for both TB [7] and ART [8], although a recent trial shows some benefits of the approach for ART [9]. While both TB and ART programmes use lay health workers, there is a stronger focus in the ART programme on patient rights, treatment preparation, motivation and social support [10], sometimes referred to as an 'empowerment approach'. While the need to change treatment models and to better integrate care delivery for TB and HIV/AIDS is discussed widely, the focus is generally on the health care system as a whole, and much less on the experiences or attitudes of the staff who need to implement such programmes in their clinics or communities. As in many settings, providers in South Africa are already under considerable pressure in their workplace - clinics are often crowded and understaffed, have scarce resources, and there is insufficient time for communication with patients [11-13]. This may affect providers' responses to new initiatives.

The City of Cape Town in South Africa recently piloted a new programme, based on the ART model, with the aim of improving TB treatment adherence. The aim of this paper was to explore provider perspectives of the implementation of the new TB treatment adherence model, the Enhanced Tuberculosis Adherence (ETA) programme, using the normalization process model as an analytic framework [14].

## Methods

### The Enhanced Tuberculosis Adherence Programme (ETA)

The aim of the ETA was to improve TB treatment outcomes, through using patient centred care, supported self-administration of treatment, and a team approach to care delivery. The key parts of the programme are described in Table 1 and the tasks and responsibilities of nurses and lay health workers within the programme are described in Table 2. This article complements papers reporting how the programme was developed (Atkins, S, Lewin, S, Ringsberg, KC, Thorson, A: Developing a new model of tuberculosis treatment support in Cape Town, South Africa: A qualitative process analysis, submitted); the findings of a parallel interrupted time series study of the effects of the programme [15]; and a study describing patient experiences [16].

**Table 1:** Key components of the ETA programme and of DOT

DOT	ETA
Training: Standard nurse training for nurses; 5 day training for lay DOT supporters	Training: Additional 1 day induction to the ETA for nurses; additional 3 day induction to the programme for DOT supporters (now called treatment supporters); adherence counsellor training for ex-DOT supporters, including five days of programme training and five days of counselling training

Patient is initiated onto directly observed therapy in the clinic (takes treatment once a day under supervision of the TB nurse)	Before initiating self-administered treatment, the patient is placed on directly observed therapy in the clinic for a short period (takes treatment once a day under the supervision of the TB nurse for approximately 2 weeks) to identify problems that might preclude self administration of treatment

Mode of treatment delivery: directly observed therapy	Mode of treatment delivery: self administration at home with pill counts by treatment supporter

Short information session about TB, and its treatment, given by the TB nurse	Trained lay adherence counsellor gives TB information to the participant in 3-4 counselling sessions, of half an hour each, focusing on treatment education, side effects, healthy living and adherence planning and TB and HIV

No visits are made routinely to patients' homes	A treatment supporter conducts a home visit to document the patient's home circumstances and verify their address. TB contacts, immunocompromised persons and children under 5 years in the household are also referred to the clinic for testing and vaccinations

No meeting of different role-players to discuss treatment support	Nurse, adherence counsellor and treatment supporter meet to discuss each patient's eligibility for self administration

Patient can receive DOT in the workplace, or by visiting a DOT supporter in the community	Patient can take treatment in the workplace, or at a clinic, but can also obtain a one month supply of tablets from the clinic and self-supervise their treatment

Nurse sees patient at diagnosis, for DOT, for 2/3 month sputum and at the end of treatment	Nurse sees patient at diagnosis, DOT for two weeks and, if the patient is eligible for self-administration, once per month until the end of treatment and for 2/3 month sputum and end of treatment sputum

If the patient is placed on community based DOT, s/he visits a treatment supporter once a day to receive treatment. Maximum DOT supporter caseload is 30 patients per month.	If the patient is placed on the ETA model, a treatment supporter visits the patient three times in the first week and once a week thereafter to monitor treatment taking. Maximum treatment supporter caseload is 60 patients per month.

No formal integration of family or friends into the treatment plan	Treatment "buddy" has an important role - s/he attends counselling and acts as a support and reminder to the patient. The buddy can be a friend, family member or neighbour of the patient

**Table 2:** Tasks and responsibilities within the new programme

Staff category	Tasks within the programme	Administrative responsibilities
Professional nurses	• Diagnosing smear-positive pulmonary TB • Initiating patients on treatment • Providing initial directly observed therapy at clinic • Monitoring patient adherence • Monthly weighing, taking sputum samples, assessing problems. • Sending patients to adherence counsellors for counselling. • Dealing with referrals to the clinic.	• Completing patient-held records (white cards) with calendar to indicate sputum dates, and other clinic appointments • Keeping track of which treatment supporter is responsible for which patient • Checking home assessment and counselling sheets; signing patient off for treatment self-administration

Adherence counsellors	• Informing patients of the programme • Counselling the patient and their buddy about TB, TB treatment, side effects of TB treatment, the importance of good adherence and promoting HIV counselling and testing • Reporting back to the team about patient's eligibility for the programme	• Filling in intervention register • Filling in counselling sheet • Filling in patient name, address and contact number for treatment supporters • Assigning treatment supporters to patients • Giving out home assessment forms

Treatment supporters	• Conducting home assessments, identifying TB contacts and individuals at risk of contracting TB in the home and referring children under 5 years of age to the clinic to be assessed for TB treatment or TB preventive therapy • Reporting back to clinic team meetings on home assessments • Visiting patients and conducting pill counts after patient placed out: three times in the first week and once a week thereafter until the patient completes treatment • Reporting back to team meetings on patients that experience problems with taking treatment	• Filling in home assessment forms • Filling in referral forms • Filling in forms when patients are not available or have problems with their treatment • Filling in monitoring forms, including when patient was visited and dates for sputum smear testing

Treatment buddy	• Attending 4 counselling sessions with patient • Supporting, motivating and reminding patients to maintain treatment adherence in the home	• Reporting problems that patients experience to treatment supporter or clinic

### The normalization process model

The normalization process model (NPM) is designed for understanding the processes of implementing a complex intervention, and understanding how interventions become workable and integrated into everyday work [14]. The model focuses on the "operational work people do to enact a set of practices" [17] and was chosen in order to highlight the experiences of staff in making the programme work in practice. The model also assists in making clear recommendations for future implementation. This was important as this programme was a pilot with a view to inform service decisions on whether and how to scale up the programme across the province. We anticipated that the model could provide insights regarding the factors that would lead to normalisation of the programme. The model has four main constructs as outlined in table 3 below.

**Table 3:** The Normalization Process Model

Main construct	Sub construct	Content
Interactional workability: how does the programme affect interactions between people and practices?	Congruence	What is dealt with within the interaction; what the work is; roles of each actor and the formal and informal rules governing the interaction
	Disposal of work	The effects and goals of the interactions; how disagreements are minimised; when and where goals and outcomes should occur; and shared beliefs about the meaning and consequences of the work

Relational integration: how does the programme relate to existing concepts and relationships?	Accountability	Knowledge and practices of the implementers; who has the knowledge, what contributions are required by participants and the formal and informal rules governing the distribution of knowledge
	Confidence	Beliefs about the knowledge and practice required by the programme, including agreement about the sources of authoritative knowledge and practice, beliefs about the practical utility of the knowledge and practice

Skill-set workability: how is the current division of work affected by the programme?	Allocation	Which tasks are performed by whom; including how these decisions are made, the distribution of resources, rewards linked to status and authority, formal and informal agreements about identification and appraisal of necessarily skills, and the definition and ownership of these skill-sets
	Performance	The ability of the organisation and the people within it to organise and deploy the intervention, including staff training needs; formal and informal boundaries of competence of workers; the degree of autonomy assigned to them; and how they deliver services

Contextual integration: how does the programme relate to the organisation in which it is set?	Execution	Practicalities of implementation; including funding, decisions on distribution of resources, costs and risks within the organisation; managerial decision-making on the taking up the intervention; and formal and informal mechanisms for its evaluation
	Realisation	Allocation and ownership of responsibility for implementing the intervention, including the negotiations necessary to change existing systems and practices to make new ones possible; minimising disruption and risk; and how new resources are obtained and used in practice

### Study setting

The programme was implemented in five TB clinics in Cape Town, South Africa. Each clinic was staffed by one or two professional nurses, as well as lay workers (in this study called adherence counsellors or treatment supporters), who were employed and managed by a non-governmental organisation (NGO). A doctor was in attendance once or twice a week. These facilities had caseloads of between 400 and 1800 TB patients per year. All five clinics were located in low-income communities of mainly Xhosa speaking Africans.

### Participants and sampling

All nurses (n = 6) and adherence counsellors (n = 6) working on the programme were asked to participate in interviews. All treatment supporters (approximately 85) were invited through adherence counsellors to participate in focus group discussions (FGDs). Table 4 details the interviews and FGDs conducted. These took place approximately four and nine months after the start of the intervention. Two nurses, three adherence counsellors and one group of treatment supporters were interviewed twice during the programme in order to establish whether their experience of the programme changed over time.

**Table 4:** Details of interviews and focus group discussions conducted

Clinic	Staff category	Number of participants (Male/Female)	Number of individual interviews (INT) or focus group discussions (FGD)
1	Professional nurses	0/2	4 INT
	Adherence counsellors	0/2	4 INT
	Treatment supporters	1/38	4 FGD

2	Adherence counsellors	0/1	1 INT
	Treatment supporters	0/7	1 FGD

3	Professional nurses	0/1	1 INT
	Adherence counsellors	0/1	2 INT
	Treatment supporters	0/7	2 INT

4	Professional nurses	1/1	2 INT
	Treatment supporters	0/6	1 FGD

5	Professional nurses	0/1	1 INT
	Adherence counsellors	0/1	1 INT
	Treatment supporters	0/5	1 FGD

Total		2/71	

### Data collection

Both the semi-structured interviews and FGD guides were thematic and focused on participants' perceptions of their tasks, including their views on the different parts of the programme; staff roles before and during the intervention; relationships with co-workers; and the challenges and successes of the programme. All individual interviews were conducted in English, at the participants' workplace. All except two interviews (at the nurse's request) were recorded digitally. Recordings were transcribed by a professional transcriber and checked for accuracy by the first author (SA). All FGDs were conducted in Xhosa (the local language) by an experienced FGD moderator with a background in social science at a venue convenient for treatment supporters. FGDs were transcribed and translated verbatim and checked for accuracy independently. No major discrepancies were found. In addition to interviews and FGDs, SA was present at clinics during implementation and was a participant observer in management level steering group meetings that discussed the implementation of the programme at intervention clinics.

### Analysis

The principles of thematic content analysis were followed [18]. Transcripts were initially read and re-read to gain familiarity with content. The transcripts were then transformed into condensed meaning units and coded openly. These codes were then organised under sub-categories, which were reviewed by all authors. After this, the transcripts were read again and the sub-categories refined. Sub-categories were then grouped under categories. These categories were examined and placed under headings according to the NPM [14]. As the interview guide was open-ended and did not focus on the NPM, not all of the items within the NPM were mentioned in the interviews. Issues relating to the implementation of the intervention were added, based on participant observation of steering group meetings, and knowledge of the implementation from a wider evaluation [19]. Validity in the study is increased through the use of multiple researchers from different disciplines to moderate data [20].

### Ethics

Ethical approval for the study was granted by the Ethics Committee of the Medical Research Council and the US Centers for Disease Control, Atlanta. All participants received informed consent forms which they read or had read to them. The voluntary nature of participation was emphasised, and interviews were assured of confidentiality and that their work or any benefits would not be affected by a decision not to participate. Participants in FGDs were asked to keep information within the group. All were provided refreshments, and treatment supporters were reimbursed for their transport costs (approximately R6/US$1 per person).

## Results

Table 5 provides a summary of the overall results, organised according to the constructs of the NPM (see table 3 for explanation) [14].

**Table 5:** Overview of promoting and inhibiting factors of ETA normalization

Main construct	Category	Promoting factors	Hindering factors
**Interactional workability**	**Congruence**	Clear roles	Lay health workers performing duties outside their set roles
			Lack of internalisation of the empowerment approach and patronising attitude to patients
	**Disposal**	Efficiency of work: reduced crowding, queues and easier follow-up Teamwork	Lack of trust in patients and doubts regarding patient adherence

**Relational integration**	**Accountability**	Training sessions	Nurses' non-attendance of training
		Dedicated project manager Treatment supporters' tacit knowledge	Strained relationships between staff
			Nurses questioning lay health worker abilities; loss of less literate but more experienced lay health workers
			
	**Confidence**	Patient appreciation of the programme Fewer challenges and increased confidence later in the programme, possibly due to reports of positive outcomes	Lack of initial buy-in (acceptance of the model) from nursing staff and lay health workers, based on the perception that HIV programmes cannot work for TB

**Skill-set workability**	**Allocation**	Clear allocation of tasks	Hierarchical nature of staff relations
			Late and insufficient lay health worker stipend payments
			Lay health worker attrition
	**Performance**	Hope for programme impact and reduced work	Introducing patients to the programme perceived as time consuming
		Patient reception of adherence counsellors' work	Administrative tasks were seen as time consuming and complicated
			Treatment supporter safety in the community
			Uncertainty about, and training needs for, questions about HIV/AIDS

**Contextual integration**	**Execution**	Resources allocated to the programme	Late and insufficient stipends for lay health workers
		High level management and NGO support	Lack of space within clinics for the programme
	**Realisation**	Dedicated project manager	Lack of participation from facility managers
			Lay health worker attrition

### Interactional workability: How did the ETA affect interactions between people and practices?

#### Congruence: What was the content of the work and what were staff roles within the ETA?

The main change required by the programme was a shift from the DOT approach to a more empowering form of treatment support. Clear roles had been assigned to each actor in the intervention. Despite the clear allocation of roles and tasks, both adherence counsellors and treatment supporters reported performing duties outside the programme. These included patient education in the waiting room, or directing patients in the clinic:

"*If I'm not busy, I'm always helping them there in the bench [waiting area of clinic]*" (clinic 1)

Treatment supporters noted that they felt like they were acting as family members and social workers to the patients. They did not resent this:

"...*to others we get to be mothers, sisters etc. For instance, with other patients they get to come to you to help with their family issues, as their 'big sister'*." (clinic 1)

The shift from DOT to a more empowering approach did not seem to have been internalised by nurses or adherence counsellors. For nurses, the main difference between DOT and the ETA seemed to be that the ETA programme was more community based than DOT and that patients were now responsible for their own treatment taking and were supported in this by their treatment buddies. Nurses and most adherence counsellors felt that their role had not changed much. In part, this was because adherence counsellors felt they had also counselled patients in their earlier role as DOT supporters.

Nurses sometimes exhibited a patronizing attitude towards TB patients, further suggesting that the empowerment approach was not internalised. In interviews some nurses described patients as "*unreliable and untrustworthy*", and one nurse compared TB patients to children when discussing how patients dealt with disappointments during the programme:

"*[The patient is] Like a child, you tell him you are going to buy sweets, but you don't buy it. Then they cry because they lose faith*" (clinic 3)

Some adherence counsellors also seemed to want to establish a higher status for themselves, in relation to patients, during counselling. One adherence counsellor referred to DOT as a tool with which she could ensure that patients adhered to treatment: " *I'm always reminding my patients, I've got DOTS. I've got ways to check when they...do this properly*..." (clinic 1)

In addition, treatment supporters made statements that suggested that their relationship with patients was not one of equals. For example, they mentioned that they were "*helping those who are not able to help themselves*" (clinic 3). This unequal relationship may also have been strengthened by treatment supporters conducting pill counts during visits - an adherence check required by the intervention.

#### Disposal: What did staff perceive as the goals and effects of the ETA?

Nurses and lay staff identified a number of benefits of the intervention, such as reduced crowding and queues and easier follow up. However, they were concerned about the effectiveness of the intervention. All staff categories expressed doubt, especially with regard to treatment adherence:

"*It's a concern because even though the adherence staff go and do the home visits, sometimes they report that they didn't see the client ...So I'm not really sure whether they are taking the medication at home. I mean, I can't assume, but I don't know... if they do default when they come here... and sometimes they do come here and they take the tablets and then you find it outside the gate!"*(clinic 4).

Some treatment supporters also questioned patients' adherence:

"*To add on DOTS was [better] than [the ETA] because you as a health worker knew for sure that the patient is taking the pills - now, even though there is someone responsible for the patient, there is no way of knowing*" (clinic 4).

Staff seemed to resolve their anxiety over not knowing whether the programme was effective by hoping that it would result in better results, and waiting for official reports from the health department on treatment outcomes.

Teamwork was a central idea of the programme. Most staff reported that they worked as a team, and that there were few disagreements. Data from observations indicated, however, some problems with teamwork at some clinics - for example, adherence counsellors sometimes argued about tasks given to them by nurses. Respondents reported that disagreements were resolved through discussion.

### Relational integration: How does the ETA relate to existing knowledge and relationships?

#### Accountability: What additional knowledge was required by the ETA?

The intervention required additional knowledge of the intervention processes and administration. Training sessions of varying lengths were conducted for all staff. Nurses had responsibility for the programme, and took on a supervisory role. However, not all nurses felt prepared for this task, especially new nurses and those who had not attended initial training:

" *... I think for the [lay health workers] they are doing the training, but for us nurses, I haven't been trained and I'm supposed to supervise. What am I supervising if I don't know anything about the [ETA]? " *(clinic 4)

In some clinics, the adherence counsellor became an unofficial manager of the programme, as they were the only staff member present at the clinic since the start of the programme. This strained working relationships and affected negatively the day-to-day operation of the programme.

When staff were asked who they would contact with questions regarding the programme, they mostly mentioned the dedicated project manager. In those clinics where there had been no changes in nursing staff, lay health workers reported they would take their questions to the nurse responsible for the programme. The role of the project manager may have created some difficulties in teamworking within the clinic, as nurses may have expected to be in control of implementation.

Nursing staff also seemed to doubt whether treatment supporters had received sufficient training in the ETA:

"*I think the training of the [treatment supporters] should be reinforced. It should not be a once-off ... They should come back and be assessed on how they're doing. Because it's no use you give all this information and the conversion rate or the cure rate does not reflect what we are doing*." (clinic 5)

Nurses also lamented the loss of older DOT supporters who did not have sufficient literacy skills to be part of the programme, but who had good relationships with patients and, in their view, could ensure adherence to treatment.

#### Confidence: Was the ETA credible according to staff?

The ETA was presented to staff as an adaptation of the community ART model. It was not uniformly accepted and some nurses questioned the approach. One felt that the ETA model could not work for TB as it had for HIV, as patients recognised the differences between the diseases:

".. *But they know that TB is curable and the other one is not curable*" (clinic 4)

Another was concerned about the confusion that could be caused by co-infected patients going through both the ART and ETA education sessions.

Overall, nurses and adherence counsellors were unsure whether the programme would be beneficial, although they felt that patients appreciated it:

"*I should think the patients... they like us to do this programme*" (clinic 1)

Most nurses suggested the need to refer to the official TB treatment outcomes before venturing an opinion on the effectiveness of the programme, suggesting that they were not convinced that the ETA would have the expected impact:

Interviewer: "*Do you think that the new intervention will make a difference in the outcomes?*"

Nurse: "*That I can't tell you. I hope it will because I don't know what the conversion rate is for the... This is the second quarter...So I mean, then we can make a proper assessment, and we will only have these results in October*."(clinic 5)

Staff reported fewer challenges and less uncertainty when they were interviewed nine months after the start of the programme, compared with earlier interviews.

### Skill-set workability: How is the current division of labour affected by the ETA?

#### Allocation: Which tasks belonged to whom? And who has the skills to implement the ETA?

Management allocated tasks to staff. Hierarchical relationships within the clinics remained, which created some frustration among staff. For example, nurses saw the lay health workers as their extensions in the community:

"*[we work] hand in hand...Or hand in glove!*" (clinic 1)

Adherence counsellors reported working hand-in-hand with the TB nurses. However, nurses seemed to see adherence counsellors as their subordinates and some monitored their work:

"*sometimes I would come here when she [the adherence counsellor] was interviewing a client and see how she was doing it*..." (clinic 4)

In turn, adherence counsellors seemed to see treatment supporters as reporting to them and seemed to place responsibility for the programme's success or failure on the treatment supporters:

"...*So if it didn't work then they [the treatment supporters] must know it's their baby - it's their fault that it didn't work. Yes, it is their fault because they are the ones who go to the clients. They are the ones who bring back the feedback to the clinic. Is the client taking treatment every day? What did they see? So they come back and report to us*." (clinic 2)

It seemed that treatment supporters were lowest in the pecking order, and saw their status and pay as similar to those of a lowly mine worker:

"*they [other staff and programme managers] should also listen to us even though we are not professionals. We are mine diggers here at the bottom. We are being paid a ridiculous amount of money...*" (clinic 1)

Treatment supporters seemed to resent their treatment and status at the clinic for a number of reasons. They were not allocated dedicated space in which to work within the clinic, but felt that the quality of their work was measured by their presence at the clinic. They also felt that adherence counsellors and nurses did not assist them but rather complicated their daily tasks by, for example, requiring them to attend the clinic despite their work being based in the community:

"... *it could be that you are in the same area as your next patient but you do not know that because they [the clinic staff] do not call you. That means you have to go to the clinic, and only to find out that you have to go back to the same area*..." (clinic 1)

One treatment supporter group also described how nurses and adherence counsellors embarrassed them in front of patients:

"*Our patients regard us as important people in their lives. The next thing we can't help them in any way when we get to the clinic. Instead we are treated in a very rude way*." (clinic 1)

The difficulties created by the hierarchy within the clinics centred mainly on treatment supporters: nurses and adherence counsellors seemed to have little trust in them and felt that more commitment and skills were needed on the part of treatment supporters. Though one nurse pointed out that treatment supporters deserved more credit for their work, this view was not held widely.

#### Performance: Could the staff implement the ETA?

Staff expressed some initial concerns about the programme's impact on their daily work. Although some nursing staff expressed positive feelings about the programme and thought it would lessen their workload, others noted that introducing the patient to the programme took time. All lay workers reported experiencing uncertainty initially. However, only treatment supporters complained about their work, as they saw it as more demanding than before. All cadres were concerned about the administration required:

"*Too many forms. If we change forms, though, how will we keep track?" *(Nurse, clinic 1)

Treatment supporters visiting the patients at home also encountered a number of challenges in finding patients:

*"You'd go until you develop blisters on your feet only to find out that he [the patient] failed to give the correct directions when he gave this address*..." (clinic 1)

They were also concerned about their safety in the communities because of substance abuse.

Treatment supporters also mentioned that they were not always well received by the patients or the patients' families. Some patients were frightened that their HIV status would be revealed to others, and some families were suspicious of the treatment supporters' motives.

Adherence counsellors reported that they were seen by patients as a source of information, with more time to spend with patients than nurses. Consequently, they encountered a number of issues in their counselling, such as poverty and substance abuse, for which they were not prepared. All adherence counsellors also reported the challenges of dealing with HIV in their counselling sessions:

*"The TB patients, they disclose [their HIV status]. Because if you are discussing this TB thing they also say, 'No, you must also know that I'm dually infected'. So that is why I know it is happening*." (clinic 5)

Some adherence counsellors were not sure how to deal with the patients' questions, especially on HIV. One adherence counsellor reported giving patients answers from a book she had acquired. Similarly, treatment supporters reported difficulties in managing the issue of HIV when visiting patients' homes:

*"...at times it is your patient who is HIV [positive] and now it feels like you asking him/her to tell the whole family*." (clinic 2)

Most staff expressed training needs related to programme implementation. Nurses wished for ongoing in-service training, despite the available project manager. Nurses and treatment supporters wanted increased and continuing training for lay health workers especially on administrative forms. Both adherence counsellors and treatment supporters felt that they needed training on HIV-related issues:

*"Yes we do need training, for example... clients who are on ARVs [antiretrovirals] and we are adherence supporters and we know nothing about ARVs*." (clinic 1)

### Contextual integration: How does the ETA relate to the organisation in which it is set?

#### Execution: What were the resource requirements of the ETA and what impact did this have on the programme?

Implementing the intervention demanded a reallocation of resources to the programme, with some additional funding needing to be sourced and directed through a non-governmental organisation (NGO). The organisation of payment for lay health workers, who were employed by this NGO, created some difficulties for intervention implementation. Payments were not always on time and the stipends were considered small:

*"They are robbing us, they take their time to give us our money, they make promises they can't keep, and they hardly support us with our needs. We know we are regarded as volunteers but they promised to give us little something to motivate us but ... we are struggling. We always keep up with our work because should we not they shout at us." *(clinic 3)

Treatment supporters especially resented these payment problems and, as some of the focus group discussions were conducted at the time of delayed payment, there was considerable discussion of how this impacted on their lives. Similarly, adherence counsellors reported they would work for the programme longer if the stipends were better:

*"Yes! Unless I can get another job that's also counselling but the money is better! But the way I like my job, I've got no problem."*(clinic 3)

The execution of the programme also presented some challenges at clinic level. As clinics were already crowded, facility managers could not allocate space for treatment supporters. In addition, there was not enough room for adherence counsellors at all clinics, which meant that they counselled in store rooms and filing rooms. This made adherence counsellors unhappy and also impacted on the confidentiality of counselling:

*"Like the lady [clinic staff] who was here just now. She [stores] things in the cupboard. Sometimes there's a client and maybe we are in the middle of the HIV thing, and then somebody [clinic staff] has to walk in. Now, the fear of the patient is that he wonders if this person has heard what he has just said." *(clinic 1)

#### Realization: What were the necessary modifications to practices and resources?

Participation observation of project steering group meetings revealed a lack of participation from facility managers, who organised care within the clinics. This created difficulties, especially in terms of space allocation.

A substantial new resource input was the employment of a dedicated project manager, who was responsible for ensuring the programme was running as intended in the clinics. Observation at the clinics revealed that the project manager gave 'hands on' help to staff, provided stationery and forms to clinics, and attended clinic and project meetings. The project manager provided another line of supervision for the intervention sites, and this may have impacted significantly on programme outcomes as staff may perform better when they feel they are being supported in their work.

One of the main challenges in realizing the programme on the ground was the supply of treatment supporters. Lay health workers would leave the programme because they found other employment; however, replacing them was not easy as new workers from the community could not be trained in advance as it was felt that this might create an expectation of employment. The high turnover of treatment supporters also created more work for nurses:

*"Treatment supporters. You know that they chop and change. You have one who was doing well and now she's going there... You know, you put for one person all the clients, and then you must do another list again because now you have to give all the clients to someone else. "(*clinic 3)

Changes in and shortages of treatment supporters meant that not all geographic areas requiring treatment support were covered, and that programme implementation took longer than anticipated, which frustrated the patients.

## Discussion

We found a number of issues that could have promoted or inhibited the normalisation of the intervention in these clinics. The two main issues were the lack of an empowerment approach and the extent to which teamwork was embraced. These issues need to be addressed before the programme is implemented more widely.

The poor internalisation of the empowerment approach seemed to be one of the largest implementation barriers. There seemed to be a general cautiousness among health care providers regarding buying in to the programme's principles. While there have been longstanding calls for TB services to focus more on the needs of clients [21,22], previous research has indicated that patient centred care is difficult to entrench in TB clinics [23-25]. There is some evidence that provider training can improve the patient centeredness of care [26], but it is likely that the training for this intervention was too short to effect a change in approach.

There is also some evidence from systematic reviews that an empowerment approach may have benefits for patient care [26,27], and HIV treatment based on this approach appears to achieve good outcomes [3,4]. In this setting, however, distrust and power differentials between patients and staff remained important. Discussions with patients indicated that patients may not have become active participants in the treatment process, as intended by the programme [16]. The intervention's limited effect on empowerment is also not surprising given the difficulties in relationships between TB patients and providers documented extensively elsewhere [6,28]. However, despite not achieving patient empowerment to a great degree, the programme did achieve outcomes that were not significantly different from the more coercive DOT approach [15]. It is also possible that after a longer period of training and intervention, and proof of positive impacts, the empowerment approach would be easier for staff to embrace.

Though teamwork was a major positive influence on programme implementation at some clinics, it was often tempered by hierarchical relationships [29]. As in previous research, treatment supporters felt undermined and maltreated [30] and while team members all shared a common goal, power differences remained. This hierarchy may have its roots in the hierarchy present in medicine in general [13] and may be difficult to modify. Problems with teamwork have been reported previously in similar settings [13,31].

Status differentials and hierarchical relationships within the clinic may also have contributed to high attrition rates for treatment supporters [32], and therefore have complicated programme implementation. Other possible contributors to attrition include the working conditions as well as the loss of older lay health workers, who did not satisfy the entry criteria for the new project. While the lay health workers employed in the ETA were more literate, they may have been more likely to leave in search of other employment [33]. Also, the older lay health workers may have been more skilled in maintaining caring relationships and providing the other intangible forms of support [34] that are important to patients [35].

Irregular payments to treatment supporters further contributed to difficulties in intervention implementation, and would need to be addressed if the programme were implemented more widely. Steady payments, and possibly hiring lay workers as formal employees of the health service, might assist in retaining staff. Other work suggests, though, that payment per se may be less important in reducing attrition and improving performance than ensuring that the expectations of both lay workers and programme managers, including with regard to incentives, are in alignment [36]. Investments should also be made in other areas of lay health workers' work experience, for example in their personal safety.

The normalisation of the intervention into routine clinic care may have been further hampered by the challenge of embedding an empowerment-oriented approach and by problems with teamworking, which may have resulted in dilution of the original intervention or diversions from its original aims. However, other factors also could have impacted on the normalisation of the intervention, specifically those related to the performance, execution and realization of the programme. Though staff generally coped with the impact of the programme on their work, they all identified training needs and highlighted day-to-day challenges, especially in terms of the high attrition rates for lay health workers and space constraints within the clinics. All staff also noted their difficulties in managing issues related to HIV/AIDS that were raised by patients during consultations. Not surprisingly in a context with a high rate of TB-HIV co-infection [37], patients do not compartmentalize the two diseases in the way that the health system does [38] and staff encounter issues related to HIV when discussing TB treatment. Ensuring that one carer manages both diseases could also help alleviate human resource constraints [39], and reduce duplication of effort. A positive influence to implementation was the introduction of a project manager, to whom staff turned when in need of training, stationery or general support. However, it is not clear whether the resources to employ a highly skilled manager would be made available in a wider roll-out of the programme. Overall, our data highlight the need for mentoring and supervision of staff at clinic level [40].

Overall, the NPM [14] assisted in identifying factors that affected the embedding of the programme within the clinics. The model provides a categorisation of the issues involved, which enables the development of recommendations for similar interventions. The challenge of implementing the NPM is that many of the categories within it overlap, and issues emerging from the interviews and FGDs may be closely interrelated and so may be difficult to assign to a single category. In addition, not all of the parts of the NPM could be addressed from the interview and focus group discussion data available; for example, nurses did not mention many issues related to the execution of the programme, possibly because they were not aware of decisions made at a higher managerial level. Observations therefore were used to supplement the model. Further work to develop tools and methods to assist in the use of the NPM would be helpful. It is also important to keep in mind that staff may have used the interviews and focus group discussions as an opportunity to raise their concerns regarding the new intervention programme, and so data on promoting factors may have been less forthcoming than data on inhibiting factors. However, the critical views of the programme expressed during data collection suggest that most participants were comfortable in expressing their opinions to the researcher.

Although staff highlighted many challenges, it is interesting to note that all clinics opted to continue with the ETA programme after conclusion of the pilot, suggesting that staff had formed positive opinions of the new approach. Further research needs to be conducted, however, to establish whether the effects of the programme are sustainable without the intensive monitoring systems used in the pilot. While these results have been obtained from a specific setting in South Africa, the implications of the findings could be applied more widely in settings with similar conditions. Table 6 includes a number of recommendations for further implementation.

**Table 6:** Recommendations concerning wider implementation of the programme

Construct	Recommendation
Interactional workability	More emphasis and training on the empowerment approach Increased attention to teambuilding

Relational integration	Increased training and supervision on the implementation of the programme and regular feedback on programme progress

Skill-set workability	Increased attention to issues related to hierarchical relationships within the clinics, and how problems caused by this could be dispelled Streamlining of administrative relationships

Contextual integration	Attention to management structures, payment systems, facilities and space for the intervention Attention to lay health worker supply and attrition

## Conclusion

Given the high rate of TB-HIV co-infection, the use of a more empowerment oriented community approach instead of DOT may assist in providing more patient centred treatment and paving the way for the better integration of TB and HIV care. Our results emphasise the need to plan carefully a major programme shift such as this. The NPM has assisted in categorising the issues impacting on efforts to shift TB treatment support from DOT to a patient centred programme. Though the intervention under study experienced a number of challenges, it may, with more development, training and attention to teamwork, present a treatment support option for TB that could be integrated more easily with the ART model.

## List of abbreviations

ART: antiretroviral treatment; DOT: Directly Observed Therapy; ETA: Enhanced Tuberculosis Treatment Adherence Programme; HIV: Human Immunodeficiency Virus; NPM: Normalization Process Model; SA: Salla Atkins; TB: Tuberculosis

## Competing interests

The authors declare that they have no competing interests.

## Authors' contributions

SA and SL conceptualised the study. SA, SL, KCR and AT all contributed to data analysis, and to editing the manuscript. SA prepared the main manuscript. All authors read and approved the final manuscript.

## Authors information

SA has a PhD in international health and was employed at the South African Medical Research Council at the time of this study. SL has a PhD in medical sociology and is employed at the South African Medical Research Council and the Norwegian Knowledge Centre for the Health Services. KCR has a PhD in social medicine and is employed as a professor of public health at the Nordic School of Public Health, Sweden. AT is a medical doctor and has a PhD in global health and is employed at Karolinska Institute, Sweden. SA, SL and AT have a longstanding interest in tuberculosis treatment models.

## Pre-publication history

The pre-publication history for this paper can be accessed here:

http://www.biomedcentral.com/1472-6963/11/275/prepub
